# Assessing social support impact on depression, anxiety, and stress among undergraduate students in Shaanxi province during the COVID-19 pandemic of China

**DOI:** 10.1371/journal.pone.0253891

**Published:** 2021-07-23

**Authors:** Kun Guo, Xiaoye Zhang, Simin Bai, Halimatus Sakdiah Minhat, Ahmad Iqmer Nashriq Mohd Nazan, Jianan Feng, Xiuqin Li, Guihua Luo, Xiaoping Zhang, Jujun Feng, Yingbo Li, Mingyu Si, Youlin Qiao, Jing Ouyang, Suhainizam Saliluddin

**Affiliations:** 1 Department of Community Health, Faculty of Medicine and Health Sciences, University Putra Malaysia, Kuala Lumpur, Malaysia; 2 College of Humanities and Management, Shaanxi University of Chinese Medicine, Xianyang, Shaanxi Province, China; 3 Medical Experiment Center, Shaanxi University of Chinese Medicine, Xianyang, Shaanxi Province, China; 4 School of Public Health, Chinese Academy of Medical Sciences & Peking Union Medical College, Beijing, China; 5 Department of Cancer Epidemiology, National Cancer Center/National Clinical Research Center for Cancer/Cancer Hospital, Chinese Academy of Medical Sciences and Peking Union Medical College, Beijing, China; Uniformed Services University of the Health Sciences, UNITED STATES

## Abstract

Following the 2019 coronavirus disease (COVID-19) outbreak in China, undergraduate students may experience psychological changes. During emergency circumstances, social support is an important factor influencing the mental health condition among undergraduate students in Shaanxi province. This study aims to find the factors associated with mental health symptoms of depression, anxiety, and stress among undergraduate students in Shaanxi province during the COVID-19 pandemic in China. A cross-sectional study was conducted from Feb 23 to Mar 7, 2020. A total of 1278 undergraduate students from the universities located in Shaanxi province participated in this study. The mental health symptoms were measured by 12-item Perceived Social Support Scale (PSSS) and Depression Anxiety Stress Scale (DASS-21) instruments. This survey showed that females receive more social support compared to males (t = -5.046, P<0.001); males have higher-level depression symptoms (t = 5.624, P<0.001); males have higher-level anxiety symptoms (t = 6.332, P<0.001), males have higher-level stress symptoms (t = 5.58, P<0.001). This study also found participants who have low social support was negatively correlated with mental health symptoms. In Conclusion, Males and low social support were associated with having the higher level of depression, anxiety, and stress symptoms among undergraduate students in Shaanxi province during the COVID-19 pandemic in China. Therefore, it is suggested that people should supply more social support for undergraduate students in Shaanxi province during COVID-19 pandemic.

## Introduction

In emergencies, it is common for individuals to feel more stressed and worried. In recent years, there have been several public emergencies in China, such as the severe acute respiratory syndrome (SARS) in 2003 [1], the natural disaster of the Wenchuan earthquake in 2008 [2], and the human avian influenza A (H7N9) virus infection in 2013 [3]. Based on experience, a large number of people will suffer from mental disorders soon after these unexpected events.

The new coronavirus pneumonia (NCP, also called COVID-19) started an outbreak as unknown etiology than was first reported in Wuhan city which is the capital of Hubei province in December 2019 [4]. The COVID-19 caused by the SARS-CoV-2 virus progressed into a pandemic in China and other parts of the world [5]. In January 2020 the World Health Organization (WHO) declared the outbreak of the COVID-19 to be a public health emergency of international concern [6]. Rapidly increasing cases and local community transmission occurred in multiple countries, including European countries and the United States [7]. In March 2020, WHO announced the COVID-19 can be characterized as a pandemic [8]. During the COVID-19 pandemic, the population may have experienced some known risk factors, such as high morbidity and mortality rate, food insecurity, discrimination, and contact with infected individuals. To reduce the morbidity and mortality of COVID-19, the Chinese government took some measures to contain the epidemic, such as the authority of Wuhan city suspending public transport indefinitely on January 23, following which similar measures were adopted soon in many other cities in China [9]. During the COVID-19 pandemic, the Chinese government closed public places such as schools and universities and mass gatherings were not allowed [10]. All the undergraduate students were instructed to stay at home with their family members in isolation for a long time.

In China, the undergraduate degree course requires four or five years of study, according to the major (e.g. medical majors require 5 years) [11]. Most of the undergraduate students are aged 18–23 years. Undergraduate students are young, healthy, have great mobility, and like to socialize, making up one of the most dynamic groups in China [12]. The COVID-19 outbreak puts the entire educational system in unprecedented difficult situations, as following the government’s requirements of “nonstop teaching and learning”, most of the undergraduate students have to continue their study online [13]. Students often have problems such as lack of self-discipline, suitable learning materials, or good learning environments when they are self-isolated at home [14]. Loss of face-to-face connections and traditional social activities cab become a stressful phenomenon for these students [15]. Therefore, it is necessary to pay particular attention to the mental health condition (anxiety, depression, stress) among undergraduate students in China during the COVID-19 pandemic.

Social support refers to “the family members, friends and others (neighbors and community members, et al) that is available in times of need to give psychological, physical, financial or other support” [16]. Previous studies have shown that depression, anxiety, and other various physical and mental symptoms were undesirable consequences after disaster [17]. After disaster, people with low levels of social support have high levels of stress with high rates of mental health morbidity and mortality [18]. Social support may also help people enhance resilience in times of emergency.

Shaanxi province with 38.64 million population which is located to the northwest of the Hubei Province and is adjacent to it. By February 7, 2020, 195 confirmed cases and 539 suspected cases of novel coronavirus pneumonia in Shaanxi Province were detected [19]. The COVID-19 outbreak occurred during winter vacation for the universities in China, so undergraduate students return to their hometown and stay with their families to celebrate the Chinese spring festival. Thus, undergraduate students formed an important part of the massive transportation utilization, which may increase the risk of being infected by COVID-19. This study aims to find the factors associated with mental health symptoms (depression, anxiety and stress) among undergraduate students studying in Shaanxi province during COVID-19 pandemic.

## Methodology

### Study design

This study was a cross-sectional survey that conducted during the period from Feb 23 to Mar 7, 2020. In this time, the Chinese government have implemented measures to control the COVID-19 pandemic. We recruited undergraduate students from 8 universities (including both public and private university) in Shaanxi province. Participants must be able to speak and comprehend Mandarin. All the participants were above 18 years old and signed the informed consent approving the use of their data for research purposes. The inclusion criteria were for the participants to be registered as a student studying in Shaanxi province and agreeing to participate in the study. Data were collected through Wenjuanxing (www.wjx.cn) with an anonymous, self-rated questionnaire that was distributed to the participants through the internet. Participants were asked to complete a set of questionnaires which included background information, the perceived social support scale (PSSS), and the Depression Anxiety Stress Scale (DASS-21). This study was approved by the Ethics Committee of Jining medical college (ref. no. JNMC-2020-KY-001).

### Measures

#### The Depression, Anxiety, and Stress Scale-21(DASS-21)

The Chinese version of Depression, Anxiety and Stress scale 21-item (DASS-21) was used to assess undergraduate students’ level of depression, anxiety and stress [20]. Previous studies have proven that DASS-21 has good construct validity and content validity [21]. DASS-21 is a globally popular self-reported scale consisting of 21 items (including three subscales of depression, anxiety, and stress, each of which contains seven items) for assessing an individual’s emotional symptoms for the past week. Each item consists of four statement, and assigns a score from 0 to 3 (0, did not apply to me at all; 1, applied to me to some degree, or some of the time; 2, applied to me to a considerable degree, or a good part of time; 3, applied to me very much, or most of the time). Overall, the psychometric construct of the Chinese-DASS-21 showed satisfactory conformity to the psychometric construct of the English version of DASS-21 (depression: item 3, 5, 10, 13, 16, 17, 21; anxiety: item 2, 4, 7, 9, 15, 19, 20; and stress: item 1, 6, 8, 11, 12, 14, 18) [22].

The manual of DASS-21 outlines that an individual’s levels of depression, anxiety, and stress are based on each subscale’s score [23]. For Depression, the criteria were normal (0–9 points), mild (10–13 points), moderate (14–20 points), severe (21–27 points), and extremely severe (28+ points). For Anxiety, the standards were normal (0–7 points), mild (8–9 points), moderate (10–14 points), severe (15–19 points), and extremely severe (20+ points). For Stress, the criteria were normal (0–14 points), mild (15–18 points), moderate (19–25 points), severe (26–33 points), and extremely severe (34+ points).

#### Social support scale

The Chinese version of 12-item Perceived Social Support Scale (PSSS) is a measure of how an individual perceives the social support level from family, friends and others [24]. Previous study had shown that the 12-item perceived social support scale had good construct validity and content validity [25]. Participants were asked to rate on their agreement to the statements in the questionnaire pertaining to their situation in the past month on a 1–7 scale, with 1 = strongly disagree and 7 = strongly agree. This measure is calculated by summing the respondent’s score across all 12 items, with scores ranging from 12 to 84, with a higher score indicating higher perceived social support.

#### Data analysis

The data were analyzed using the IBM SPSS Statistics for Mac (version 26). Descriptive analysis was used to describe the general data. T-test was used to compared with the mean value of the continuous variables. Pearson correlation analysis was compared the variables. For the determination of independent predictors for depression, anxiety, stress of undergraduate students in Shaanxi province during the COVID-19 pandemic in China, the odds ratio (OR) was estimated based on multivariate logistic regression analysis. An OR of less than 1 was associated with a lower likelihood of experienced depression, anxiety, and stress during the COVID-19 pandemic, while an OR of greater than 1 was associated with a higher likelihood of experienced depression, anxiety, and stress during the COVID-19 pandemic. P values < 0.05 indicated that had a significant statistically. The significance level was set as α = 0.05 (two-tailed) in all data analysis.

## Result

### Descriptive statistics in each variable

A total of 1275 questionnaires were collected and 452 males and 826 females were included in the study. Table 1 shows the demographic data and the level of mental health symptoms of undergraduate students. In Table 1 showed that 280 (21.9%) undergraduate students had depression symptoms; 270 (21.1%) had anxiety symptoms; 138 (10.8%) had stress symptoms; 12 (0.9%) respondents had low social support score; 461(31.6%) had moderate social support score; 805 (63%) had high social support score. The means, standard deviations, minimum values, and maximum values for the DASS-21 and PSSS are shown in Table 2. Furthermore, the gender differences in each variable are presented in Table 2 showing different gender have different depression level (t = 5.624; P<0.001); different gender have different anxiety level (t = 6.332; P<0.001) and different gender have different stress level (t = 5.58; P<0.001). The different gender obtained different level of “support from family”(t = -3.704; P<0.05); the different gender obtained different level of “support from friends” (t = -4.85; P<0.001); the different gender obtained different level of “support from others” scores (t = -5.37; P<0.05); the different gender obtained different level of “total social support” (t = -5.046, P<0.001).

**Table 1:** Undergraduate student demographic characteristics and the level of mental health symptoms. **DOI:** 10.6084/m9.figshare.13106753.

Characteristic	n	Percentage (%)
**Age**		
<21	712	55.7
21–22	232	18.2
>22	334	26.1
**Gender**		
male	452	35.4
female	826	64.6
**Class year**		
First year	358	28
Second year	348	27.2
Third year	274	21.4
Fourth year	159	12.4
Fifth year	139	10.9
**social support**		
low support	12	0.9
moderate support	461	36.1
high support	805	63
**Depression**		
normal	998	78.1
mild	96	7.5
moderate	123	9.6
severe	31	2.4
extremely severe	30	2.3
**Anxiety**		
normal	1008	78.9
mild	55	4.3
moderate	122	9.5
severe	29	2.3
extremely severe	64	5
**Stress**		
normal	1140	89.2
mild	49	3.8
moderate	47	3.7
severe	37	2.9
extremely severe	5	0.4

**Table 2:** Descriptive statistics of each variable by gender and gender differences in each variable.

			n	Mean	SD	Minimum	Maximum	SE	df	t	95%CI	P
DASS-21	Depression	Males	452	6.78	8.52	0	42	0.4	1276	5.624	(1.51,3.12)	<0.001***
		Females	826	4.47	6.07	0	38	0.21				
	Anxiety	Males	452	5.81	8.18	0	42	0.38	1276	6.332	(1.65,3.13)	<0.001***
		Females	826	3.41	5.28	0	28	0.18				
	Stress	Males	452	7.15	8.63	0	42	0.406	1276	5.58	(1.54,3.2)	<0.001***
		Females	826	4.78	6.38	0	34	0.22				
Social support	Family	Males	452	21.15	4.61	4	28	0.22	1276	-3.704	(-1.42,-0.44)	0.003**
		Females	826	22.08	4.11	6	28	0.14				
	Friend	Males	452	20.92	4.5	4	28	0.21	1276	-4.85	(-1.59,-0.68)	<0.001***
		Females	826	22.06	3.71	6	28	0.13				
	Others	Males	452	20.13	4.34	4	28	0.2	1276	-5.37	(-1.71,-0.80)	0.004**
		Females	826	21.39	3.8	7	28	0.13				
	Total support	Males	452	62.2	12.53	12	84	0.59	1276	-5.046	(-4.62,-2.03)	<0.001***
		Females	826	65.52	10.51	19	84	0.37				

Notes: Gender Differences were calculated by two independent samples t-test. DASS-21 = the Chinese version of Depression, Anxiety and Stress scale 21-item; Family = Support from family; Friends = Support from friends; Others = Support from others.

***p<0.001

**p<0.01.

DOI:10.6084/m9.figshare.13106756

### Correlations among all variables

Results of correlation analysis are shown in Table 3. Age had positive significantly correlations with class year (r = 0.776, p<0.001), and age had negative significantly correlations with social support (r = -0.056, p<0.05). Social support had negative significantly correlations with depression (r = -0.282, p<0.001), anxiety (r = -0.249, p<0.001), and stress (r = -0.245, p<0.001). Therefore, the results show that social support are negative significantly correlated with depression, anxiety, and stress.

**Table 3:** Correlation matrix of the factors and mental health condition of undergraduate students. **DOI:**10.6084/m9.figshare.14806536.

	1	2	3	4	5	6	7
1.age	1						
2.gender	-0.012	1					
3.class year	0.776**	0.093**	1				
4.social support	-0.056*	0.174**	-0.009	1			
5.depression	0.037	-0.156**	0.007	-0.282**	1		
6.anxiety	0.028	-0.168**	-0.007	-0.249**	-0.282**	1	
7.stress	0.041	-0.154**	0.015	-0.245**	0.882**	0.902**	1

Note

**p<0.001

*p<0.05.

### Predicting mental health symptoms

In Table 4 showed that males were more likely to have depression symptoms than females (OR = 0.639, P<0.05); males were more likely to have anxiety symptoms than females (OR = 0.627, P<0.05); males were more likely to have stress symptoms than females (OR = 0.551, P<0.05). Participants who have low social support were likely to have higher level of depression symptoms (OR = 0.104, P<0.001). Participants who have low social support were likely to have higher level of anxiety symptoms (OR = 0.096, P<0.001). Participants who have low social support were likely to have higher level of stress symptoms (OR = 0.14, P<0.001). Participants who have moderate social support were likely to have lower depression symptoms (OR = 0.359, P<0.001). Participants who have moderate social support were likely to have lower level of anxiety symptoms (OR = 0.401, P<0.001). Participants who have moderate social support were to have lower level of stress symptoms (OR = 0.441, P<0.001) (Table 4).

**Table 4:** Multiple logistic regressions with factors and social support predicting mental health symptoms. **DOI:** 10.6084/m9.figshare.14806545.

Variables	Depression			Anxiety			Stress		
	β	OR (95% CI)	P	β	OR (95%CI)	P	β	OR (95%CI)	P
**Age**									
<21	0.174	1.19(0.707–2.001)	0.512	0.248	1.282(0.748–2.196)	0.366	-0.001	0.990(0.51–1.921)	0.976
21–22	-0.064	0.938(0.575–1.531)	0.798	0.186	2.204(0.715–2.208)	0.484	0.256	1.292(0.666–2.504)	0.449
>22	reference								
**Gender**									
Male	-0.448	0.639(0.480–0.850)	0.002	-0.467	0.627(0.468–0.840)	0.002	-0.596	0.551(0.379–0.8)	0.002
Female	reference								
**Class year**									
First year	0.297	1.346(0.680–2.665)	0.393	0.069	1.071(0.532–2.155)	0.847	0.498	1.646(0.673–4.026)	0.275
Second year	0.257	1.293(0.681–2.457)	0.432	0.13	1.139(0.588–2.206)	0.7	0.014	1.014(0.445–2.312)	0.974
Third year	0.559	1.749(0.984–3.109)	0.057	0.549	1.731(0.956–3.133)	0.07	0.418	1.52(0.715–3.228)	0.276
Fourth year	0.72	2.054(1.133–3.723)	0.018	0.789	2.201(1.738–4.111)	0.013	0.392	1.48(0.682–3.209)	0.321
Fifth year	reference								
**Social support**									
Low support	-2.263	0.104(0.03–0.357)	<0.001	-2.342	0.096(0.028–0.332)	<0.001	-1.967	0.140(0.042–0.471)	<0.001
Moderate support	-1.024	0.359(0.271–0.476)	<0.001	-0.914	0.401(0.300–0.535)	<0.001	-0.819	0.441(0.682–3.209)	<0.001
High support	reference								

## Discussion

The prime objective of this study was to find the factors associated with the mental health symptoms of anxiety, depression, and stress among undergraduate students in Shaanxi province during COVID-19 epidemic in China. The mean age of the participants in this study was 21.46±1.68 years, 35.4% of them males and 64.6% of them females. In this study there were more female than male respondents; this corresponds with findings from a similar study carried out among university students in Nigeria [26]. From the response questionnaire frequency of this research found the response rates vary by gender, with women being more likely to respond than men [26].

The results of independent-samples t test indicate that females perceived higher level of total social support (support from family, friends and others) compared to males. This result is consistent with previous studies and the study recognized that females have more support resources than males in general, because females tend to construct diverse interpersonal networks [27]. This reason was due to gender differences in self-disclosure. CL Tam revealed that females become involved in more in-depth communication through willingness to express their problems and stress, and females also had larger social networks, more sources to draw support, and are more satisfied with friends compared to males [28]. In this study, a significant difference for perceived support from family between gender was found, which was contrary to prior findings that females perceived less support from family than males [29]. This result may be due to most of the undergraduate students are not married, and the support from their family continues as usual during the COVID-19 pandemic in China. Therefore, females get more support from family, friends and others due to the fact that generally people acknowledge that females need more protection than males [30].

The analysis of mental health symptoms between genders revealed significance. Males have higher level mental health symptoms of depression (t = 5.624, p<0.001), anxiety (t = 6.332, p<0.001) and stress (t = 5.58, p<0.001) than females in this study. This result was contrary to some previous research indicating that females appeared to bear a greater burden of mental distress than males, such as in Alexandria and Egypt, where the rate of having depressive symptoms in girls was almost double that of boys; however, there was no significant difference in Oman [31]. In this study, males have higher levels of anxiety, depression and stress which may be explained by the weaker social support for males. This result may be due to males should take on more social responsibilities in Chinese society. A previous study on the mental health of the general population during the COVID-19 epidemic in China found that they have moderate-to-severe anxiety, depression and stress of 28.8%, 16.5% and 8.1%, respectively [32]. In this study, undergraduate students in Shaanxi province have moderate-to-severe anxiety, depression and stress with prevalence of 16.8%, 14.3% and 7% respectively. Compared to the mental health of general population during COVID-19 epidemic in China, undergraduate students in Shaanxi province have less anxiety which may be caused by undergraduate students having access the COVID- 19 information online quickly.

This research design focused on discovering the relationship between social support and mental health symptoms among undergraduate students in Shaanxi province. Previous study also found the negative relationship between social support and anxiety, depression and stress symptoms [33], but our study is the first study focus on the social support and mental health symptoms among undergraduate students of Shaanxi province during the COVID-19 pandemic in China. Our study results were contrary with the findings reported by Y. Zhang and Ma ZF. who investigated social support as being positively related with mental health symptoms during COVID-19 pandemic in China [4]. One possible reason for getting social support is related to mental health symptoms among undergraduate students during COVID-19 pandemic in China is that all the undergraduate students were back home and staying with their family members to celebrate Chinese spring festival. Chinese spring festival as the beginning of a new year has an important significance for a fresh start and hope for good things to come. During the Chinese spring festival, there is increased communication with family members and family members were more likely to care for each other and spend time together. During COVID-19 pandemic, if undergraduate students perceived more social support will avoid to have higher level of depression, anxiety, stress symptoms occurring.

This study also has some limitations. Firstly, the nature of cross-sectional study design means it was unable to determine causality between the variables. Secondly, we collect data online that may have bias we can’t control. Thirdly, our study enrolled 8 university in Shaanxi province that might not reflect the actual situation of undergraduate students in Chinese universities as a whole. Despite these limitations, this study still presents the depression, anxiety and stress situation related to the COVID-19 outbreak. Identifying the factors associated with depression, anxiety, and stress situation of the COVID-19 outbreak is important to find which factor can affect depression, anxiety and stress symptoms among undergraduate students in Shaanxi province of China. From this study, it was found that gender and social support level were associated with depression, anxiety, stress symptoms among undergraduate students in Shaanxi province, which provide the valuable reference for future studies on mental health condition.

To our knowledge, this study is the first investigation assessing the social support and the mental health symptoms among undergraduate students in Shaanxi province during COVID-19 pandemic in China. Therefore, it provides valuable insights in the network and mental health condition. Our results indicate that social support can influence mental health symptoms directly among undergraduate students during the COVID-19 pandemic in China. In order to maintain a good mental health for undergraduate students, public health management should encourage supply more social support to undergraduate students.

## Conclusion

In conclusion, this study found females get more support from family, friends and others, and males had higher level of depression, anxiety and stress symptoms than females. There are negative correlations between social support and mental health symptoms among undergraduate students in Shaanxi province during COVID-19 epidemic in China. Therefore, it is suggested that people should supply more social support for undergraduate students in Shaanxi province during COVID-19 pandemic.

## Supporting information

S1 Questionnaire (DOCX)
