# Supplementary material for: Assessing social support impact on depression, anxiety, and stress among undergraduate students in Shaanxi province during the COVID-19 pandemic of China
Source: PLoS One. 2021 Jul 23;16(7):e0253891. doi: 10.1371/journal.pone.0253891 (PMC8301624; doi:10.1371/journal.pone.0253891)
Supplement: S1 Questionnaire — (DOCX) [file pone.0253891.s001.docx]

**A survey of social support and mental health symptoms among undergraduate students in Shaanxi province during the COVID-19 pandemic of China**

1. If you agree to participate in this survey, please check the "I agree to participate in this survey" option; if you do not agree, please check the "Disagree" option, and your interests will not be affected in any way:

○ I agree to participate in this survey ○ Disagree (skip to the end of the questionnaire)

I、**Basic Information:**

| 1. Have you been quarantined at home or at designated points by your school, work unit or community before?  ○ YES ○No |
| --- |
| 2．Have you infected by the COVID-19?  ○ YES ○No  3. Date of your confirmed or suspected infection: ________(year)______(month).  4. Your confirmed or suspected infection location is: ________(province)______(city).  5. Please specify your gender:  ○Male ○Female  6. Data of birth： ________year ______month |
| 7. Please specify your race.  ○Han ○Hui ○Zang ○Weiuer ○Zhuang  ○Menggo ○Others |
| 8.The current city is ________(province)______(city). |
| 9. Please specify your class year:  ○1 ○2 ○3 ○4 ○5 |
| 10.The subject you study:  ○ Social ○ Science ○Medical |

**II.** Perceived social support scale (PSSS）

|  | Very strongly disagree（1） | | strongly disagree  （2） | | Mildly disagree  （3） | Neutral  （4） | Mildly Agree  （5） | Strongly Agree（6） | Very Strong Agree（7） |
| --- | --- | --- | --- | --- | --- | --- | --- | --- | --- |
| 1.There is a special person who is around when I am in need. | | □ | | □ | □ | □ | □ | □ | □ |
| 2.There is a special person with whom I can share my joys and sorrow. | | □ | | □ | □ | □ | □ | □ | □ |
| 3.My family really tries to help me. | | □ | | □ | □ | □ | □ | □ | □ |
| 4.I get the emotional help and support I need from my family. | | □ | | □ | □ | □ | □ | □ | □ |
| 5.I have a special person who is a real source of comfort to me. | | □ | | □ | □ | □ | □ | □ | □ |
| 6.My friends really try to help me. | | □ | | □ | □ | □ | □ | □ | □ |
| 7.I can count on my friends when things go wrong. | | □ | | □ | □ | □ | □ | □ | □ |
| 8.I can talk about my problems with my family. | | □ | | □ | □ | □ | □ | □ | □ |
| 9.I have friends with whom I can share my joys and sorrows. | | □ | | □ | □ | □ | □ | □ | □ |
| 10.There is a special person in my life who cares about my feelings. | | □ | | □ | □ | □ | □ | □ | □ |
| 11.My family is willing to help me make decisions. | | □ | | □ | □ | □ | □ | □ | □ |
| 12.I can talk about my problems with my friends. | | □ | | □ | □ | □ | □ | □ | □ |

III. **Depression-Anxiety-Stress Scale 21, DASS-21**

Please read each statement and circle a number 0, 1, 2 or 3 which indicates how much the statement applied to you over the past week. There are no right or wrong answers. Do not spend too much time on any statement.
The rating scale is as follows: 0 Did not apply to me at all-NEVER
1 Applied to me to some degree, or some of the time - SOMETIMES
2 Applied to me to a considerable degree, or a good part of time - OFTEN 3 Applied to me very much, or most of the time - ALMOST ALWAYS

|  | N (0) | S (1) | O (2) | AA  (3) |
| --- | --- | --- | --- | --- |
| 1.I found it hard to wind down. | □ | □ | □ | □ |
| 2.I was aware of dryness of my mouth. | □ | □ | □ | □ |
| 3.I couldn’t seem to experience any positive feeling at all. | □ | □ | □ | □ |
| 4. I experienced breathing difficulty (eg, excessively rapid breathing, breathlessness in the absence of physical exertion) | □ | □ | □ | □ |
| 5. I found it difficult to work up the initiative to do things | □ | □ | □ | □ |
| 1. I tended to over-react to situations | □ | □ | □ | □ |
| 7.I experienced trembling (eg, in the hands) | □ | □ | □ | □ |
| 1. I felt that I was using a lot of nervous energy | □ | □ | □ | □ |
| 9. I was worried about situations in which I might panic and make a fool of myself | □ | □ | □ | □ |
| 1. I felt that I had nothing to look forward to | □ | □ | □ | □ |
| 1. I found myself getting agitated | □ | □ | □ | □ |
| 1. I found it difficult to relax | □ | □ | □ | □ |
| 1. I felt down-hearted and blue | □ | □ | □ | □ |
| 14. I was intolerant of anything that kept me from getting on with what I was doing | □ | □ | □ | □ |
| 15. I felt I was close to panic | □ | □ | □ | □ |
| 16. I was unable to become enthusiastic about anything | □ | □ | □ | □ |
| 17. I felt I wasn’t worth much as a person | □ | □ | □ | □ |
| 18. I felt that I was rather touchy | □ | □ | □ | □ |
| 19. I was aware of the action of my heart in the absence of physical exertion (eg, sense of heart rate increase, heart missing a beat) | □ | □ | □ | □ |
| 20. I felt scared without any good reason | □ | □ | □ | □ |
| 21. I felt that life was meaningless | □ | □ | □ | □ |
